# Nutritional Life Cycle Assessment of Cow Milk in Northern Italy: Implications for Comparisons with Plant-Based Alternatives

**DOI:** 10.3390/foods15142541

**Published:** 2026-07-18

**Authors:** Federico Froldi, Maurizio Moschini, Antonio Gallo, Alessia Moroni, Lucrezia Lamastra, Filippo Rossi, Riccardo Negrini, Margherita Dall’Asta, Erminio Trevisi

**Affiliations:** 1 Department of Animal Science, Food and Nutrition (DiANA), Università Cattolica del Sacro Cuore, 29122 Piacenza, Italy; federico.froldi@unicatt.it (F.F.); maurizio.moschini@unicatt.it (M.M.); antonio.gallo@unicatt.it (A.G.); alessia.moroni@unicatt.it (A.M.); filippo.rossi@unicatt.it (F.R.); riccardo.negrini@unicatt.it (R.N.); erminio.trevisi@unicatt.it (E.T.); 2 Romeo and Enrica Invernizzi Research Center for Sustainable Dairy Production of the Università Cattolica del Sacro Cuore (CREI), 29122 Piacenza, Italy; 3 Department for Sustainable Food Process (DiSTAS), Università Cattolica del Sacro Cuore, 29122 Piacenza, Italy; lucrezia.lamastra@unicatt.it; 4 Invernizzi Reference Center on Agri-Food (IRCAF), Università Cattolica del Sacro Cuore, Campus Santa Monica, 26100 Cremona, Italy

**Keywords:** functional units, carbon footprint, nutritional composition, environmental impacts, dairy

## Abstract

This study evaluated the environmental impact of milk production in Northern Italy using a nutrient life cycle assessment (nLCA) approach with different functional units (FUs) related to the nutritional value of whole milk at the farm gate. Data from 94 dairy farms in the production areas of two protected designations of origin (PDOs) were analyzed. The FUs considered were based on milk mass, protein, calcium, essential amino acids (EAA), portion size, and milk energy content. Climate change (CC) impact was assessed using Intergovernmental Panel on Climate Change (IPCC) guidelines. The assessment estimated environmental impacts of 0.17, 4.78, 0.14, 1.06, 0.21, and 0.33 kg CO_2_-eq for the FUs: 100 g of milk, 100 g of protein, 100 mg of calcium, 10 g of EAA, 125 mL portion size, and 100 kcal of milk, respectively. Results showed no significant differences in CC between PDOs and geographical areas (plain vs. mountain) for any selected FU. However, when comparing cow milk with plant-based alternatives, product ranking changed substantially depending on the selected FU: while milk showed the highest impact per 100 g product, its relative performance improved when expressed per 100 g protein. These findings demonstrate that FU choice influences the interpretation of environmental results.

## 1. Introduction

The UN estimates that food demand will double by 2050 due to population growth, requiring a 50–70% increase in agricultural productivity, particularly in developing countries where food security remains a challenge [1,2,3]. In this context, milk and dairy products represent a key food group supporting human nutrition [4]. Milk is a nutrient-dense food, with a relatively low energy density that provides significant amounts of high-quality proteins and micronutrients, including calcium, magnesium, vitamin A, and B12, although it is composed of approximately 87% water. Milk and dairy products represent the fifth largest source of energy and the third largest source of protein and fat globally, and they are key foods for meeting the recommended nutrient levels [3]. Milk and low-fat dairy products are key components of several dietary patterns, and the evidence suggests that dairy consumption is predominantly associated with neutral or protective associations with adverse health outcomes [5]. In Italy, food-based dietary guidelines recommend the daily consumption of milk and low-fat dairy products, with particular emphasis on the role of milk and yoghurt in helping individuals meet the daily reference values for calcium and protein [6,7]. Despite the well-recognized role of milk in supporting human nutrition, there are also potential negative aspects in milk production to be highlighted [8], such as environmental impacts related to greenhouse gas emissions (GHG), land use, water depletion, eutrophication, and acidification of water bodies, among the main ones. Among these impact categories, climate change is one of the most widely reported indicators in dairy LCA studies and is frequently used to assess the environmental performance of milk production systems [9,10]. Regarding the dairy sector supply chain, raw milk production at the dairy farm phase is the major source of emissions affecting the sector’s environmental performance [10,11]. Management systems, farming type, milk yield, inputs used, and milk quality are among the factors that influence the environmental performance of dairy farms, as they are linked to productivity levels and resource use efficiency [12,13,14,15]. These factors may vary substantially across production systems (i.e., intensive vs. extensive or plain vs. mountain systems), potentially leading to differences in environmental performance [11]. Assessing whether such variability exists within Northern Italian dairy systems is relevant for farmers and stakeholders, because differences in environmental performance may inform management strategies and sustainability-oriented decision-making at the farm level [16,17]. Although the agricultural and agri-food sectors have contributed to reducing hunger, improving life expectancy, and decreasing global poverty, several contradictions remain regarding food production systems [18]. At the same time, the increasing prevalence of the double burden of malnutrition and related non-communicable diseases highlights the need to ensure healthy diets within sustainable food systems [19,20]. Therefore, environmental and nutritional dimensions should be integrated into a holistic approach to support more sustainable agri-food development. An applicable approach is life cycle assessment (LCA) [21,22], which is an ISO-standardized method for estimating the environmental impact of products and processes [23,24,25]. However, environmental impacts and nutritional aspects of foods have often been evaluated separately rather than within a single integrated framework. Therefore, there is increasing interest in developing nutritional life cycle assessment (nLCA) approaches that integrate environmental impacts with the nutritional value of foods [26]. LCA should be based not only on the mass or volume of food but also on functional units (FUs) that reflect the different nutritional components of complex food matrices [27]. In dairy LCA studies, FUs are commonly based on fat- and protein-corrected milk (FPCM) to facilitate comparisons between farms [28]. However, this approach mainly reflects productive performance and does not fully account for the nutritional characteristics of milk, which limits comparisons with alternative foods or beverages [29,30]. This aspect is particularly relevant when comparing foods with different nutritional profiles [26]. In recent years, several studies have explored this methodological approach [31,32,33] without identifying a unified approach. The scientific literature reports studies that have focused on FUs that include a single nutrient or multiple nutrients through nutritional indices [34], depending on the study’s aims [35]. To address concerns related to milk production and support the transition toward healthier and more sustainable diets, increasing attention has been paid in recent years to plant-based milk alternatives (PBMAs) [36], which are increasingly consumed as substitutes for cow milk in many countries, such as soy milk and almond milk [37]. These products are becoming increasingly popular in Europe and other industrialized countries [38] as well as in Italy [37], because consumers often perceive them as nutritionally equivalent substitutes for cow milk. However, their composition and nutritional value may differ substantially depending on ingredients and formulation. The most consumed PBMAs are oat, almond, and soy drinks, followed by coconut and rice drinks [38]. These plant-based alternatives are often considered more sustainable than cow’s milk, although LCA studies show that their environmental performance varies by impact category [39]. As a consequence, replacing cow’s milk with PBMAs may lead to differences in nutrient intake, particularly in calcium and high-quality protein [39]. Recent studies have compared the environmental impacts of cow milk and PBMAs, generally reporting lower GHG for PBMAs when expressed on a mass basis, with smaller differences when impacts are evaluated on a protein basis [40,41]. However, the high variability in the composition of PBMAs, due to differences in ingredients and formulations, leads to product-specific outcomes, making it difficult to draw general conclusions when comparing PBMAs with cow milk [42,43].

Based on these premises, this study aimed to jointly assess (i) the variability of environmental impacts across different dairy production systems in Northern Italy, (ii) the influence of different nutritional FUs on the interpretation of these impacts, and (iii) the implications of these methodological choices when comparing cow milk with PBMAs. This integrated approach is relevant because production systems affect environmental performance, whereas FUs shape how these impacts are interpreted when cow milk is compared with nutritionally dissimilar substitutes. It was hypothesized that climate change impacts would differ among dairy production systems, particularly between plain/intensive and mountain/extensive farms, due to differences in herd management, feeding strategies, pasture use, and input intensity. It was also hypothesized that the choice of FU would affect the comparison between cow milk and PBMAs, with mass-based and protein-based FUs leading to different product rankings.

## 2. Materials and Methods

This study considered data from 94 dairy farms in the production areas of two dairy-protected cheese designations of origin (PDOs) in Northern Italy: Grana Padano (GP) PDO and Asiago (AS) PDO. The selected farms and their respective activity data constituting the life cycle inventory (LCI) are derived from the specific PDOs’ Made Green in Italy environmental certification studies [44,45]. These studies were compiled with the Product Environmental Footprint Category Rules (PEFCR) for dairy products [46] to assess the environmental impact of the respective supply chains of the two PDOs, thus representing the dairy production system in Northern Italy. The LCI reported in this study (Table 1) was simplified from the original inventories of the PDOs. The environmental impact of milk production (cradle-to-farm-gate) was evaluated using an nLCA approach, focusing on CC and applying different FUs. To enable a comparison with PBMAs commonly available on the market and widely discussed in the literature, beverages derived from soy, rice, and almonds were selected for the study. To assess the impact, the Su-EATABLE LIFE (SEL) database [47] was selected to extract the mean values of the environmental impact (kg CO_2_-eq).

### 2.1. Farm Boundary and Life Cycle Inventory

The dairy farm samples were classified according to geographical area and breeding systems, that is, 11 and 6 mountain (M) dairy farms (access to pasture-extensive system) and 56 and 21 plain (P) dairy farms (confined-intensive system) for GP and AS PDO, respectively (Table 1). The mountain herds had access to summer pasture for 2–4 months, assisted by hay during dry periods. Lowland farms provided a confined breeding system with occasional access to outdoor paddocks for the animals to rest. To ensure consistency in the modelling of seasonal management systems, quantitative and qualitative data collection referred to one production year. Grazing and confinement periods were integrated within the annual LCI. Enteric fermentation emissions were calculated based on annual dry matter intake, accounting for seasonal diet differences, while manure management emissions distinguished between direct deposition during grazing and storage and field application during confinement (Table 2).

The original LCIs were produced through several direct interviews with farmers to gather primary and secondary data. The data refer to the years 2020 and 2022 for GP and AS PDOs, respectively. The primary data flow system was modelled into different categories at the farm boundaries (Table 1): herd management data (number of animals reared and farmed area), in-farm feeds (main crop categories produced and their inputs), off-farm feeds (main feed categories purchased), other inputs (main inputs for farm management), and milk data (amount and quality). Secondary data refer to the production of chemical fertilizers, pesticides, seeds, energy and other inputs related to dairy farms.

Activity data emissions were estimated according to the IPCC [48] and EEA [51,53] guidelines (Table 2).

### 2.2. Functional Units

A product’s FU plays an important role in providing an objective and measurable reference point for attributing input and output factors [54]. To correctly define FU, specific requirements must be met, as outlined by Hauschild et al. [21] and the European Commission [55].

In this study, several FUs at the dairy farm gate were analyzed based on the nutritional value of milk, including 100 g of milk, 100 g of protein, 10 g of essential amino acids (EAA), 100 mg of calcium, portion size (125 mL), and energy (100 kcal). The protein content was determined using a Milko Scan FT6000 (Foss Electric, Hillerød, Denmark), whereas calcium content was retrieved from the CREA food composition database [56]. The EAA content was estimated based on the CREA database according to the protein content of each analyzed sample. The portion size was defined according to the reference values reported for the Italian population [57]. Energy (100 kcal) was calculated based on protein, fat, and lactose content using the conversion factors provided in Regulation (EU) No 1169/2011 [58]. The nutritional values used to define the FUs were as follows: energy 68.0 (68.0–70.0) kcal/100 g; protein 3.4 (3.3–3.5) g/100 g; EAA 1.55 (1.51–1.58) g/100 g (expressed as 25th–75th percentile). The calcium content was set at 119 mg/100 g based on the CREA food composition database. For the comparison with PBMAs, beverages derived from soy, rice, and almonds were considered. In this case, the comparison was limited to FUs based on mass (100 g product) and protein content (100 g protein) due to data availability constraints and the high variability in micronutrient composition across products. Although other nutritional indicators, such as calcium and EAA, could provide additional insights, consistent and comparable data for these parameters were not available for the selected PBMAs, which would have limited the robustness of the comparison. To ensure comparability with PBMAs marketed in the same geographical area as the milk analyzed, nutritional information was retrieved from Angelino et al. [37], which reports median values for energy and nutrient contents of the most available PBMAs sold in Italy. It should be noted that the environmental impact values for PBMAs were obtained from literature-based datasets and may reflect system boundaries different from the cradle-to-farm-gate approach adopted for milk production in this study (Table S1).

### 2.3. Allocation

The selection of the allocation approach is a controversial issue in LCA studies because of its significant impact on the results [59]. The dairy sector is characterized by multifunctionality in food production, that is, the ability to generate multiple and different outputs [60]. In this study, impacts were allocated between milk and meat using a mass allocation approach, in accordance with ISO recommendations [25] and the International Dairy Federation guidelines [28]. The allocation was based on the relative mass of milk and meat produced at the farm level, considering meat derived from culled cows and calves sold immediately after birth or after rearing. This approach allows environmental impacts to be consistently distributed between milk and meat production [61], with, on average, more than 86% of the impacts allocated to milk.

### 2.4. Estimating Impact Assessment

Life cycle inventory analysis was conducted using the SimaPro^®^ 9.5.0.0 LCA software tool developed by Pré Sustainability B.V. [62]. Ecoinvent database v. 3.9.1 was used as the background database to model upstream processes and provide detailed input and output data (elementary flows) for the analyzed system [63,64,65]. The CC indicator expresses the environmental impact of milk according to IPCC [66] guidelines, using a 100-year global warming potential. Climate change was selected as the reference impact category because it is one of the most widely used indicators in dairy and food LCA studies and allows the results to be expressed consistently across the selected nutritional FUs. This choice also enabled a focused exploratory assessment of how FU selection affects the interpretation of environmental impacts. The characterization factors for the main GHG were considered as follows: 1 kg of CO_2_-eq/kg CO_2_, 27 kg of CO_2_-eq/kg CH4—biogenic, 29.8 kg of CO_2_-eq/kg CH4—fossil, land use-transformation, 273 kg of CO_2_-eq/kg N_2_O.

### 2.5. Statistical Approach

The normality of the data was examined using the Shapiro–Wilk test, and the data satisfied the assumptions required for parametric inferences. Accordingly, a parametric analytical approach was adopted. A two-factor analysis of variance (ANOVA) was conducted to assess the influence of PDO (GP vs. AS), geographical area (P vs. M), and their interaction on the CC indicators associated with milk production. CC was assessed in multiple FUs (Table 3). The statistical model referred to the main effects of the PDO and geographical area, as well as their interaction (PDO × geographical area). The analysis was performed using JMP Pro version 17.0.0 software (SAS Institute Inc., Campus Drive, Cary, NC, USA). Statistical significance was set at *p* < 0.05.

## 3. Results

### 3.1. Climate Change Impact on the Milk Samples

**Figure 1:**
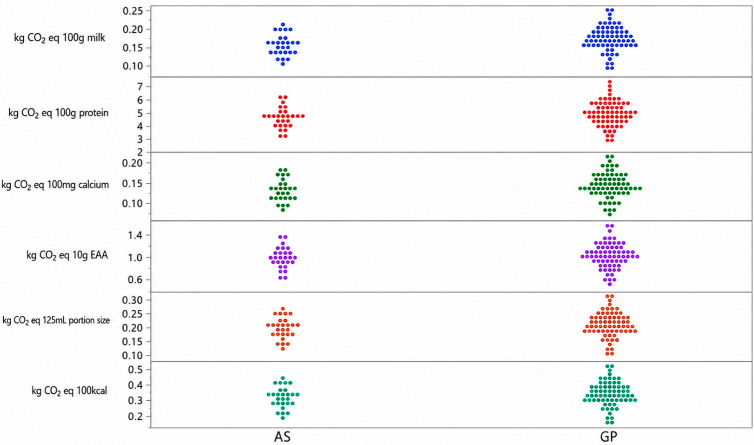
Comparative climate change impact of milk from Asiago PDO (AS) and Grana Padano PDO (GP) across multiple nutritional LCA functional units. Different color represents different functional units.

**Figure 2:**
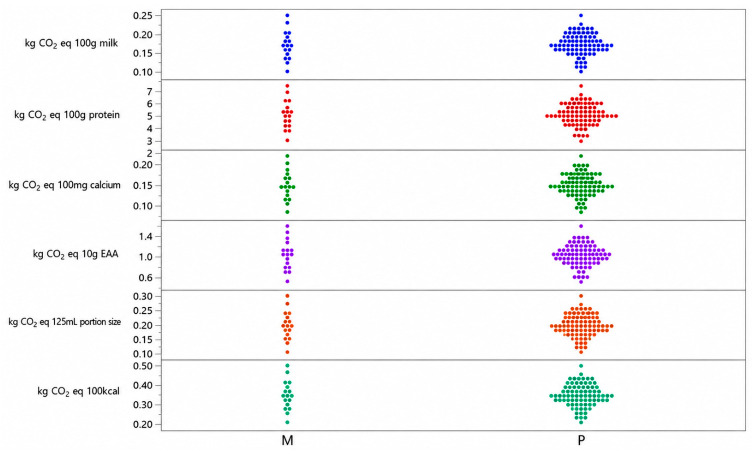
Comparative climate change impact of milk from mountain (M) and plain (P) areas across multiple nutritional LCA functional units. Different color represents different functional units.

The total number of milk samples considered for assessing on-farm milk quality was derived from fortnightly bulk milk analyses, yielding a total of 2256 samples. The CC assessment showed environmental impact values of 0.17, 4.78, 0.14, 1.06, 0.21, and 0.33 kg CO_2_-eq, corresponding respectively to the FUs of 100 g of milk, 100 g of protein, 100 mg of calcium, 10 g of EAA, a 125 mL portion size, and 100 kcal of milk (Table 3). To assess potential differences between systems, a two-factor ANOVA was performed considering PDO, geographical area, and their interaction. No significant differences in CC were observed between PDOs, geographical areas, or their interaction for the selected FUs (Table 3). Effect estimates and 95% confidence intervals are reported to support interpretation. The comparative climate change impacts by PDO and geographical area are shown in Figure 1 and Figure 2, respectively.

### 3.2. Comparison of Cow Milk and Plant-Based Alternatives

**Figure 3:**
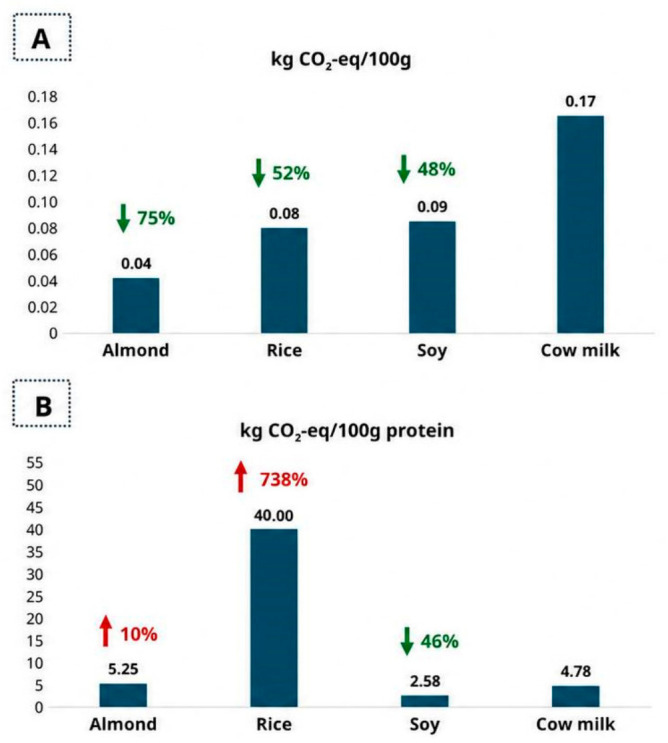
Comparison among PBMAs (Almond, Rice and Soy drinks) and the AS and GP milks in terms of kg CO_2_-eq/100 g of products (**A**) and in terms of kg CO_2_-eq/100 g of protein (**B**). Red and green arrows indicate % of increase and decrease, respectively, compared to cow milk.

A comparative analysis of the impact of CC based on a selected nutritional FU (protein content) of cow milk and plant-based alternatives was conducted. Figure 3A,B show the impact of the mass and protein content, respectively, of the analyzed milk samples in comparison with that of PBMAs. The protein contents of the plant-based alternatives were 3.3 (3.0–3.6) g/100 g for soy drink, 0.2 (0.0–0.4) g/100 g for rice drink, and 0.8 (0.5–1.0) g/100 g for almond drink. In terms of kg CO_2_-eq/100 g (3A), milk had the most impact on CC, doubling the values of soy and rice drinks, whereas almond drink had the least impact (approximately 76% less than milk). When a different FU was tested (100 g protein), the ranking of products changed substantially (Figure 3B). In this case, rice-based drinks had the highest impact and variability, with a median of 40 kg CO_2_-eq/100 g of protein (range: 20-undefined, since the 25th percentile of protein content was 0), approximately eight times higher than that of milk and almond beverages, whose median impacts were 4.78 (4.71–5.00) and 5.25 (4.2–8.4) kgCO_2_-eq/100 g of protein, respectively. Soy drinks had the lowest impact, with a value of approximately half that of milk, specifically 2.58 (2.36–2.83) kg CO_2_-eq/100 g of protein.

## 4. Discussion

### 4.1. Variability of Environmental Impacts Across Production Systems

The environmental impact of milk production in Northern Italy was evaluated across different FUs with respect to the CC impact indicators. In LCAs of food items, selecting the FU is crucial and can significantly influence the interpretation of the results [67]. However, it is important to distinguish between the role of FUs and the underlying drivers of environmental impacts, as FUs influence how impacts are expressed and interpreted, whereas emission levels are primarily determined by farm management practices and production characteristics. Considering the analysis of multiple FUs, the observed CC impacts were comparable across different PDOs and geographical areas. The absence of statistically significant differences between PDOs and geographical areas should not be interpreted as evidence that the systems are environmentally equivalent or homogeneous. Rather, it indicates that, within the available dataset and modelling framework, between-system differences were not large enough to be detected for the selected CC indicators. The unbalanced group sizes, particularly the limited number of mountain farms, may have reduced the statistical power to detect differences among production systems. For this reason, PDO and geographical area should be interpreted as exploratory grouping variables used to assess potential variability among relevant Northern Italian dairy production contexts, rather than as evidence of clear environmental differentiation among systems. The overall dataset therefore remains important for supporting the main nLCA interpretation and the comparison with PBMAs. At the same time, this result may also partly reflect relatively comparable management conditions across the analyzed systems. In this context, impacts expressed on a mass basis are broadly comparable with values reported in the literature, whereas the use of nutritional FUs mainly affects the interpretation of these impacts rather than their absolute magnitude. Nevertheless, the limited availability of detailed compositional data may further constrain the application of more refined nLCA approaches, such as those based on farm-level milk quality data. However, differences in farm management and feeding strategies have been shown to influence environmental impacts [11,68], and variability across production systems has been widely reported in the literature [17,69,70]. In the present study, the relatively narrow confidence intervals observed across FUs suggest limited variability and consistent environmental performance among the analyzed systems. A comparison with the scientific literature showed that the CC impact of the analyzed FUs was lower than that reported by McLaren et al. [35]. These differences may be attributed to the level of detail in the inventory data used in the studies [71], including the use of primary milk composition data, as well as to geographical context and differences in diets, seasons, and breeds [21,72]. In addition, the dispersion of results observed within each FU reflects variability in farm management practices rather than the choice of FU itself, with greater heterogeneity in plain systems compared to mountain systems, likely due to differences in feeding strategies and input intensity.

### 4.2. Effect of Functional Units and Data Limitations in nLCA

As previously stated, milk standard quality analyses are limited to variables of routine milk analyses, such as protein, fat, lactose, total bacterial count, and somatic cell count (SCC). However, how to represent the nutritional value of food products in LCA studies remains an open question. In this context, the choice of FU influences the interpretation of environmental results rather than the underlying emissions, particularly when nutritional FUs are applied to complex food matrices. Nonetheless, as Cassarino et al. [73] stated, excluding some nutrients rather than others represents a critical limitation in nLCA analysis. This implies that selecting specific nutrients (i.e., protein or calcium) may bias the interpretation of results, as foods provide multiple nutritional functions simultaneously. While food composition databases provide general information on the nutritional content of milk [74], these data are typically based on average values and do not reflect farm-specific variability related to feeding strategies and management systems [15,68]. This limits the application of nLCA approaches at the farm level, where detailed compositional data would be required. Milk provides nutrients with high biological value, including high-quality proteins and essential micronutrients [75,76,77,78,79]. Nonetheless, the absence of detailed and standardized compositional data at the farm level, together with the lack of harmonization in nLCA approaches, makes it difficult to compare results across different studies [80]. Regarding portion size, differences observed in the literature depend on the selected reference amount. In this study, a portion size of 125 mL was adopted according to national dietary guidelines [57], whereas other studies have used larger portions (i.e., 250 mL) [35]. As portion size represents a consumption-based FU reflecting dietary recommendations rather than intrinsic product characteristics, its use may introduce variability unrelated to production systems and limit comparability across studies. These aspects highlight that the choice of FU is not neutral, but strongly shapes the interpretation of environmental results [21], particularly when comparing products with different nutritional profiles, and underline the need for improved data collection systems capable of linking environmental and nutritional information at a finer resolution.

### 4.3. Main Drivers of Environmental Impacts in Dairy Systems

To better interpret the environmental hotspots identified in the study, the main drivers influencing the environmental impacts of milk production are discussed. Although a formal sensitivity analysis of milk production was not performed, the main processes affecting environmental outcomes can be inferred from the modelling framework and the LCI adopted in this study. The datasets used were derived from two certification studies [44,45]. At the farm level, feed production represents a major contributor to environmental impacts due to the use of inputs such as fertilizers, energy, and water [11]. Enteric fermentation is another key source of GHG emissions, while manure management contributes through CH4 and N_2_O released during storage and field application [16]. Variations in feed management strategies, herd productivity, manure handling practices and energy use can significantly influence the environmental performance of dairy production systems [17] and help explain the variability observed among the farms analysed in this study. In particular, differences in feed management strategies (i.e., purchased vs. on-farm feed production) may contribute to the observed variability, reflecting structural differences between production systems [81,82]. These findings suggest that management-related factors play a key role in shaping environmental outcomes [10,70], even when overall differences between systems are not statistically significant, and confirm that the variability observed is primarily driven by production practices rather than methodological choices according to Berton et al. [69].

### 4.4. Comparison with Plant-Based Milk Alternatives

These considerations help contextualize the comparison with PBMAs. Previous studies have shown that milk generally has a higher environmental impact than plant-based alternatives when expressed on a mass basis [38,80]. In particular, differences of approximately 57–70% have been reported between milk and plant-based beverages such as almond, oat, and soy drinks [38]. However, as shown in the Section 3: Results, this comparison changes when impacts are expressed using nutritional FUs. From a dietary perspective, the comparison between milk and PBMAs strongly depends on the FU considered [83]. When impacts are expressed per quantity of product, milk presents higher environmental impacts, whereas expressing impacts per unit of protein leads to a different interpretation, highlighting the importance of considering the nutritional contribution of foods [73]. In line with previous findings, milk showed lower impacts than some plant-based beverages when normalized per unit of protein, for example, being approximately 60% less impactful than oat drinks, while remaining more impactful than soy drinks (by approximately 70%). Almond-based beverages showed higher variability, with impacts in some cases exceeding those of milk by more than 100%, reflecting differences in formulation and protein content. Overall, these results are consistent with those reported in the literature [38,41], although differences may arise due to variability in datasets, product formulations, and methodological assumptions [47]. Variability across PBMAs is strongly influenced by differences in protein content and formulation [84]. Because impacts are normalized per unit of protein, beverages with lower protein concentrations require larger quantities of product to deliver equivalent nutritional value, which contributes to the observed dispersion in results. As previously reported by Angelino et al. [37], high variability in nutritional composition can be observed depending on the production process and specific product formulation. In particular, differences in the proportion of the main plant ingredient, the use of added sugars or oils, and the presence of micronutrient fortification may substantially influence the nutritional profile of PBMAs. These differences may therefore explain the variability observed in the environmental impacts when results are expressed through nutritional FUs. Importantly, these findings highlight that comparisons between cow milk and PBMAs should not rely solely on mass-based metrics, especially when these products are used as substitutes in the diet [85]. In such cases, differences in nutritional composition, particularly protein content and quality, should be explicitly considered to ensure a meaningful interpretation of environmental impacts [36,37,80]. This also suggests that the choice of FU may substantially influence the perceived sustainability of alternative products. Therefore, the practical relevance of protein-based FUs is mainly related to comparisons among nutritionally different products, such as cow milk and PBMAs, rather than to discrimination of dairy production systems within the present dataset.

### 4.5. Methodological Implications and Limitations

Although expressing impacts per unit of protein allows a comparison of beverages on a nutritional basis, a more comprehensive analysis should also consider other nutritional components. Calcium was not included because the nutritional composition data from Angelino et al. [37] were derived exclusively from the information reported on food labels in accordance with Regulation (EU) No. 1169/2011 [58], under which calcium is not part of the mandatory nutrition declaration. We selected the dataset by Angelino et al. because it provides a representative overview of the nutritional composition of plant-based milk alternatives available on the Italian market. Consequently, calcium values were available only for products bearing a nutrition claim related to calcium (e.g., “source of calcium”), as permitted by Regulation (EC) No. 1924/2006. These products had a median calcium content of 120 mg/100 mL, whereas calcium content was unavailable for products without such claims. Therefore, a calcium-based functional unit could not be consistently applied across all selected PBMAs. Including only fortified products, or integrating calcium data from other sources, would have compromised the representativeness of the Italian market dataset and introduced selection bias. Our results confirm the importance of considering the nutritional contribution of foods, particularly their protein content, in relation to environmental impacts, as well as the need to carefully select appropriate FUs. These aspects should also be interpreted within the context of the overall diet. In European populations, protein intake is generally adequate [86], suggesting that the consumption of beverages with lower protein content does not necessarily compromise nutritional adequacy [87]. In this context, PBMAs with lower protein concentrations may still be compatible with a balanced diet, particularly when consumed as part of a diversified dietary pattern rather than as a direct one-to-one replacement of cow milk. Therefore, the relevance of nutritional FUs depends on the dietary context in which foods are consumed. When PBMAs are used to fully replace cow milk, differences in nutrient composition become more critical, whereas their implications may be more limited when these products are integrated within an already nutritionally adequate diet. This highlights the importance of complementing product-based assessments with diet-level considerations when interpreting the environmental and nutritional implications of food substitutions [88,89]. In this perspective, there is a need to better explore the integration of nutritional aspects into the LCA approach, which requires efforts to create a dedicated database that includes both the complete nutritional composition and quality attributes of dairy products. Such databases should include comprehensive information on macro- and micronutrient composition, amino acid profiles and nutrient bioavailability, ideally linked to specific production systems. One specific aspect to be considered is the nutritional quality parameters (such as the digestible indispensable amino acid score [DIAAS]), which are crucial for better describing the nutritional contribution of food products in nLCA assessments.

This study had several strengths and limitations. First, this study reports a high number of milk samples analyzed for the main characteristics (protein, fat, and lactose content). However, more detailed information on amino acid composition and protein digestibility was not available at the farm level, which prevented the inclusion of protein quality indicators in the assessment. Second, the milk samples considered were representative of a specific, yet highly productive geographical area. The limitations of this study include the restricted and limited range of FUs considered due to the unavailability of data on micronutrients, which could allow the use of more complex and comprehensive nutritional indices to describe the overall nutritional value of food products (i.e., Nutrient Rich Food index). Another limitation concerns the application of nLCA to food products: in this study, the integration of nutritional FUs was performed using farm-gate data without accounting for the processing steps applied before milk reaches the consumer at the industrial level. This could represent a limitation, although the stage with the highest impact on milk is primary production, with an average of 94% [11], which was considered in this study. Finally, because PBMA impacts were sourced from secondary datasets, these comparisons should be interpreted as indicative and contextual rather than as a fully harmonized life cycle comparison.

## 5. Conclusions

This study aimed to describe the environmental impact of representative dairy farms in Northern Italy by exploring the use of selected nutritional FUs. No differences were observed between the different production systems or geographical areas in Italy, suggesting low inter-sample variability in emissions across all the FUs considered. As expected, milk presented a higher environmental impact when assessed on a mass basis. However, when protein content was used as the FU, the interpretation of the impact profile changed significantly compared with other PBMAs. This resulted in a relatively lower environmental burden of cow milk when expressed per unit of protein, particularly in scenarios where PBMAs are used to fully replace cow’s milk despite having a different nutritional profile. From a farm-management perspective, the results indicate that the use of nutritional FUs does not replace the need to act on the main production drivers of emissions. Therefore, mitigation strategies should continue to prioritize improvements in feed efficiency, herd productivity, manure management, and energy use, as these factors directly affect the climate change impact of milk production.

This observational study highlights the importance of considering nutrient quantity when quantifying and comparing the environmental impact of food products or food categories. Therefore, it is desirable to define and use FUs that better reflect the nutritional contribution of a product when assessing its environmental impacts. From a public policy perspective, the presented findings support the need for sustainability-oriented dietary recommendations that consider both environmental impacts and nutritional contributions, rather than relying exclusively on mass-based indicators. This is particularly relevant when comparing nutritionally different foods and when adapting dietary guidance to different socioeconomic contexts, including both high-income and developing countries. Harmonized databases integrating environmental impacts with nutrient composition and quality are needed to support more balanced and evidence-based recommendations. Moreover, future studies are needed to better explore the use of more comprehensive FUs, which should be extended to other dairy categories beyond the farm gate.

## Figures and Tables

**Table 1:** Average inventory data of dairy farms.

	Dairy Farm Management											
	GP PDO						AS PDO					
	Total		P		M		Total		P		M	
Inventory	Mean	± SD	Mean	±SD	Mean	±SD	Mean	±SD	Mean	±SD	Mean	±SD
Farms, n	67		56		11		27		21		6	
Herd data												
Dairy cows, farm	129	140	146	147	46	36	66	76	72	85	46	23
Other animals ^1^, n	146	156	165	164	51	48	57	88	62	99	42	23
Crop arable land, ha	37	59	45	62	1	3	35	39	35	42	33	33
Grassland, ha	15	25	11	23	36	26	9	18	9	19	7	10
In-farms feeds	Input data											
Cereals ^2^, t	36	113	43	123	-	-	74	175	58	133	137	306
Forages ^3^, t	1741	2347	2036	2462	240	207	560	1226	733	1374	405	409
Protein feeds ^4^, t	1	3	1	3	-	-	5	15	3	7	13	30
Diesel, lt	28,448	33,800	32,684	35,487	6885	4611	12,806	20,389	13,451	21,885	10,546	15,422
Irrigation water, m^3^	137,527	228,568	164,541	241,177	-	-	51,691	106,659	56,448	118,835	35,038	47,339
Chemical fertilizers ^5^, kg N	4185	8361	5004	8929	13	42	1337	3023	1034	1808	2397	5719
Off-farms feeds												
Cereals ^2^, t	144	190	164	197	44	101	32	55	33	59	26	41
Forages ^3^, t	152	205	172	217	52	53	22	92	27	104	6	16
Protein feeds ^4^, t	90	147	103	154	25	73	22	68	20	70	30	63
Compound feeds ^6^, t	251	528	278	572	113	104	144	200	156	216	102	134
Other inputs												
Water used on farm, m^3^	8199	9209	9425	9589	1956	1639	3568	4987	3975	5568	2141	1468
Electricity, kWh	61,405	64,953	70,088	67,592	17,202	12,052	26,914	32,162	28,797	35,564	20,325	15,897
Milk data	Output data											
Average milk yield, t	1415	1617	1578	1692	586	777	711	1123	786	1261	450	298
Protein; %	3.43	0.12	3.43	0.12	3.38	0.11	3.44	0.12	3.45	0.12	3.38	0.10
Fat, %	3.95	0.17	3.96	0.15	3.86	0.22	3.95	0.24	4.01	0.20	3.75	0.27

^1^ Dry cows, heifers, young heifers and calves. ^2^ Mais flour, maize flakes, wheat bran, barley flour. ^3^ Corn silage, wheat silage, barley silage, sorghum silage, alfalfa, polyphyte hay. ^4^ Soybean meal, soy flakes, sunflower meal, cotton seeds. ^5^ Urea (46%), ammonium nitrate (27%), N-P-K (15-15-15), N-P-K (32-0-18). ^6^ Compound feeds referred to lactating cows, dry cows, heifers, young heifers, and calves.

**Table 2:** Emissions estimated and methods used for milk production.

Emissions	Substances	Methods of Reference
Enteric fermentation	CH_4_—air	Tier 2 [48]
Manure storage, pre-treatment and application	CH_4_—air	Tier 2 [48]; Regional Legislative Decree X/5171 [49]; Regional Legislative Decree X/5418 [50]
	Direct N_2_O—air	Tier 1 [48]; Regional Legislative Decree X/5171 [49]; Regional Legislative Decree X/5418 [50]
	Indirect N_2_O—air	Tier 2 [48]
	Indirect N2O—leaching	Tier 2 [48]; Regional Legislative Decree X/5171 [49]; Regional Legislative Decree X/5418 [50]
	NH3 and NOx—air	Tier 2 [51]; Regional Legislative Decree X/5171 [49]; Regional Legislative Decree X/5418 [50]
Nitrogen fertilizer application	NO_3_—groundwater	Tier 1 [48]
	Direct N_2_O—air	Tier 1 [52]
	Indirect N_2_O—air	Tier 1 [52]
	Indirect N_2_O—(N) leaching	Tier 1 [48]; Regional Legislative Decree X/5171 [49]; Regional Legislative Decree X/5418 [50]
	NH_3_ and NO_x_—air	Tier 2 [53]; Regional Legislative Decree X/5171 [49]; Regional Legislative Decree X/5418 [50]
Urea application	CO_2_—air	Tier 1 [52]

**Table 3:** Climate change expressed as kg CO_2_-eq for the specific functional units.

	Total	PDO		GA		*p*-Value			SE	95% CI (Total)
	Mean	GP	AS	P	M	PDO	GA	PDO* GA		
Milk 100 g	0.17	0.17	0.16	0.16	0.16	0.17	0.97	0.99	0.009	0.15–0.19
Milk protein 100 g	4.78	4.96	4.59	4.72	4.83	0.15	0.66	0.96	0.253	4.28–5.28
Milk calcium 100 mg	0.14	0.14	0.13	0.14	0.14	0.17	0.97	0.99	0.007	0.13–0.15
Milk EAA 10 g	1.06	1.10	1.02	1.05	1.07	0.15	0.66	0.96	0.056	0.95–1.17
Milk portion size 125 mL	0.21	0.21	0.20	0.20	0.20	0.17	0.97	0.99	0.011	0.19–0.23
Milk 100 kcal	0.33	0.34	0.32	0.33	0.33	0.17	0.97	0.99	0.018	0.29–0.37

Values are reported as means ± SE, with 95% confidence intervals (CI). *p*-values refer to the effects of PDO, geographical area (GA), and their interaction.

## Data Availability

The original contributions presented in the study are included in the article/Supplementary Materials. Further inquiries can be directed to the corresponding author.

## Supplementary Materials

The following supporting information can be downloaded at: https://www.mdpi.com/article/10.3390/foods15142541/s1, Table S1: Descriptive statistics of GHG for milk and PBMAs. Values are expressed as kg CO_2_-eq per kg of product.
