# Supplementary material for: Nutritional Life Cycle Assessment of Cow Milk in Northern Italy: Implications for Comparisons with Plant-Based Alternatives
Source: Foods. 2026 Jul 18;15(14):2541. doi: 10.3390/foods15142541 (PMC13409401; doi:10.3390/foods15142541)
Supplement: Supplementary file 1 [file foods-15-02541-s001.zip › foods-4398046-supplementary.pdf]

Table S1. Descriptive statistics of GHG for milk and PBMAs. Values are expressed as kg CO_2-eq per kg of product.

Product	N (references)	Mean	SD	Median	Min	Max	System boundary
Milk (AS+GP)	2	0.17	0.03	0.16	0.10	0.25	Cradle-to-farm
Almond drink	2	0.42	0.02	0.42	0.39	0.44	Regional Distribution Centre
Rice drink	3	0.80	0.21	0.66	0.64	1.09	Distribution Centre
Soy drink	22	0.86	0.26	0.78	0.28	1.40	Retailer (references n=9); Sale outlet (references n=3); Regional Distribution Centre (references n=4); Distribution Centre (references n=6)
